# Isolation of Recombinant Phage Antibodies Targeting the Hemagglutinin Cleavage Site of Highly Pathogenic Avian Influenza Virus

**DOI:** 10.1371/journal.pone.0061158

**Published:** 2013-04-05

**Authors:** Jinhua Dong, Akira Sakurai, Namiko Nomura, Enoch Y. Park, Futoshi Shibasaki, Hiroshi Ueda

**Affiliations:** 1 Graduate School of Science and Technology, Shizuoka University, Suruga-ku, Shizuoka, Japan; 2 Department of Chemistry and Biotechnology, The University of Tokyo, Bunkyo-ku, Tokyo, Japan; 3 Molecular Medicine Project, Department of Genome Medicine, Tokyo Metropolitan Institute of Medical Science, Setagaya-ku, Tokyo, Japan; National Institute for Viral Disease Control and Prevention, CDC, China

## Abstract

Highly pathogenic avian influenza (HPAI) H5N1 viruses, which have emerged in poultry and other wildlife worldwide, contain a characteristic multi-basic cleavage site (CS) in the hemagglutinin protein (HA). Because this arginine-rich CS is unique among influenza virus subtypes, antibodies against this site have the potential to specifically diagnose pathogenic H5N1. By immunizing mice with the CS peptide and screening a phage display library, we isolated four antibody Fab fragment clones that specifically bind the antigen peptide and several HPAI H5N1 HA proteins in different clades. The soluble Fab fragments expressed in *Escherichia coli* bound the CS peptide and the H5N1 HA protein with nanomolar affinity. In an immunofluorescence assay, these Fab fragments stained cells infected with HPAI H5N1 but not those infected with a less virulent strain. Lastly, all the Fab clones could detect the CS peptide and H5N1 HA protein by open sandwich ELISA. Thus, these recombinant Fab fragments will be useful novel reagents for the rapid and specific detection of HPAI H5N1 virus.

## Introduction

Influenza is a highly contagious disease caused by viruses that belong to the family *Orthomyxoviridae* [1], [2]. Influenza viruses are classified into 16 HA and 9 NA subtypes on the basis of two surface proteins on the virus particle, hemagglutinin (HA) and neuraminidase (NA) [3], [4], and almost all possible subtypes have been isolated from avian species [5], [6]. HA is a glycoprotein responsible for virus binding to sialic acid on the host cell surface, and mediates the fusion of the viral and cellular membranes after endocytosis [7], [8]. HA is translated as a precursor HA_0_, which is assembled into viral particles [9]. Because the cleavage of HA_0_ into HA_1_ and HA_2_ is required for the fusion of viral and cellular membranes, the expression patterns of host proteases determine the viral organ tropism [10], [11]. The HA_0_ of low pathogenic influenza viruses is cleaved by extracellular serine proteases, which exist in a limited number of cells or tissue types. However, the HA_0_ cleavage site (CS) of the highly pathogenic avian influenza (HPAI) viruses contains a multi-basic sequence, which is cleaved by ubiquitous intercellular endoproteases including PC6 and furin [10], [11], [12]. Thus, HPAI viruses are able to enter multiple cell types and organs, and cause systemic infections.

Since the first lethal human infection in 1997 in Hong Kong, HPAI H5N1 viruses have spread worldwide; they pose a major risk for a new influenza pandemic [13]. The World health organization reported that the HPAI H5N1 virus has infected 608 individuals, causing 359 deaths [14]. The *HA* gene of HPAI H5N1 virus belongs to the A/goose/Guangdong/1/96 (H5N1) lineage, and all HPAI H5N1 viruses have a characteristic multibasic sequence in the HA CS [15]. Although there is no evidence that HPAI H5N1 viruses transmit between mammals, an experimentally mutated HPAI H5N1 virus has been transmitted via droplets in a ferret model [16]. Thus, the scientific and public health communities need to prepare for a potential HPAI H5N1 pandemic.

Hence, the diagnosis and subtyping of HPAI H5N1 viruses are high priorities for public health. For detecting HPAI H5N1 virus and diagnosing influenza, a number of specific monoclonal antibodies have been developed [17], [18]. However, because the primary structure of H5N1 HA is highly homologous to H1 subtype viruses, these monoclonal antibodies might have considerable cross-reactivity [19]. In the present study, we report several unique recombinant Fab fragments obtained from an immunized phage display library that target the CS peptide of HA derived from HPAI H5N1 virus (HA331), and we discuss their potential applications in diagnostics.

## Results

### Selection of recombinant anti-HA331 Fab fragments by phage library screening

**Figure 1:**
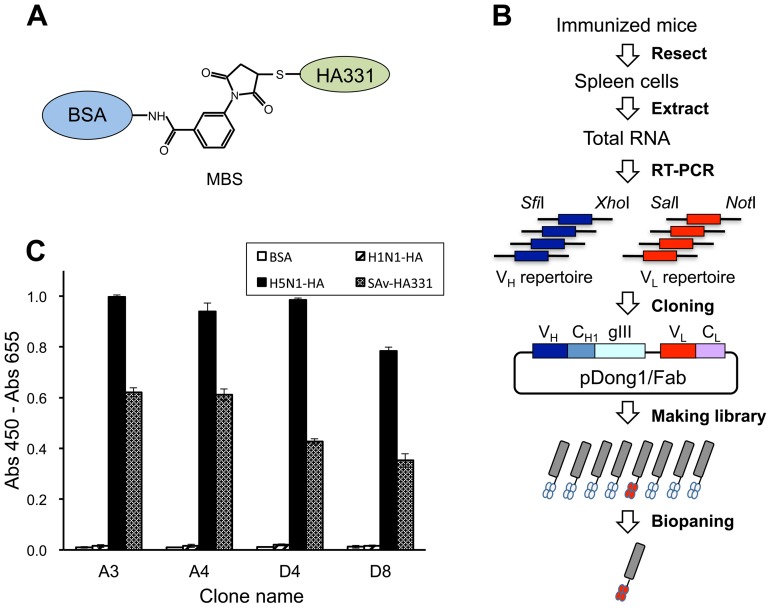
Selection of anti-HPAI HA antibodies. (A) The structure of BSA-MBS-HA331 used for mice immunization. (B) Flow chart for the development of monoclonal Fabs. The RNA extracted from the spleen cells of immunized mice was used for RT-PCR, which produced V_H_ and V_L_ cDNAs. These fragments were used to make the pDong1 phagemid library that was subsequently used for biopanning the phage libraries. (C) ELISA of the binding of positive Fab-phages to HA 331 peptide and HA proteins. HA331: cleavage site peptide; MBS: m-maleimidobenzoic acid N-hydroxysuccinimide ester; BSA: bovine serum albumin; H1N1-HA: recombinant A/California/04/2009 H1N1 HA; H5N1-HA: recombinant A/Vietnam/1194/2004 H5N1 HA.

The strategy for making anti-HA331 monoclonal antibodies is shown in Fig. 1, A and B. First, mice were immunized with the HA331-bovine serum albumin (BSA) conjugate. After the quantitation of peptide-specific antibodies in sera, the variable region genes of the antibody heavy (V_H_) and light (V_L_) chains were prepared and cloned to a phagemid vector to perform phage display selection. We used a pDong1/Fab phagemid vector that was previously used to clone anti-T4 Fab fragments [20]. Using this system, the cDNA fragments for V_H_ and V_L_ were iteratively cloned into pDong1/Fab, and a bacterial library with a diversity of 5×10^6^ was used to make the Fab-phage library. After three rounds of biopanning selection, an ELISA with immobilized HA331 peptide was performed with the original (R0) and selected (R1–R3) libraries to confirm the enrichment of HA331-specific phages. The signals for R0, R1, R2 and R3 phages increased gradually in the ELISA, confirming the enrichment of specific Fab-phages (data not shown).

### Monoclonal antibody selection

The phages obtained at round 3 were used to infect bacteria, and ninety-six clones were selected and cultivated for making Fab-phage. When an ELISA was performed, four clones—A3, A4, D4, and D8—showed strong signal against immobilized streptavidin (SAv)-HA331, and these were further analyzed. When the specificity of these clones was tested with two different HA proteins and BSA (Fig. 1C), clones A3, A4, D4, and D8 clearly bound to both the positive control, SAv-HA331, and the H5N1 HA containing a multibasic CS (A/Vietnam/1194/04). In contrast, none of the clones bound to H1N1 HA or BSA, suggesting their specificity for the H5N1 HA CS.

### Characterization of binding specificity

**Figure 2:**
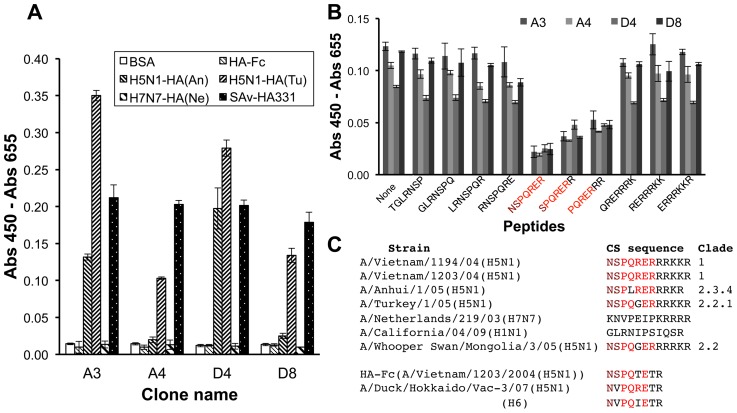
Binding specificity of the clones. (A) ELISA to determine binding to several other HA proteins. H5N1-HA(An): A/Anhui/1/05(H5N1) HA; H5N1-HA(Tu): A/Turkey/1/2005(H5N1) HA; H7N7-HA(Ne): A/Netherlands/219/03(H7N7) HA. (B) ELISA to determine epitope sequence. The inhibitory effect of 7-mer partial peptide sequences shown on the binding of Fab-phages to SAv-HA331 peptide was investigated. The peptide NSPQRER showed maximal inhibitory effect. (C) The cleavage site peptide sequence of HA proteins used. The presumptive peripheral and core epitope sequences are shown in brown and red, respectively. H6 is shown as a reference.

To further characterize the binding specificity of the obtained clones, phage ELISA was performed for several other HA proteins (Fig. 2A). As a result, clones A3 and D4 showed relatively strong binding to the two H5N1 HAs with slightly different CS, while other two clones showed weaker binding to these proteins. On the contrary, negligible binding was observed for HA-Fc whose CS is mutated, or for an H7N7-HA that has similar but distinct multibasic CS sequence (Fig. 2C).

To clarify the epitope sequence(s) recognized by these clones, epitope scanning based on phage ELISA added with overlapping 7-mer peptides was performed (Fig. 2B). In spite of lower signal due to lower titer of the phages used, the result clearly showed an asymmetric inhibition pattern involving a core sequence of (NS)PQRER for all the four clones. In other words, the core epitope sequence of the clones was not the multibasic sequence itself, but a neighboring HPAI H5N1 HA-specific characteristic sequence. However, this will be favorable for cellular diagnosis since the multibasic sequence itself will be cleaved upon viral infection. When this epitope sequence is mapped on the individual HA sequences, a clear correlation of the reactivity and amino acid identity to the immunized peptide was observed (Fig. 2C).

### Preparation and characterization of soluble Fab fragments

**Figure 3:**
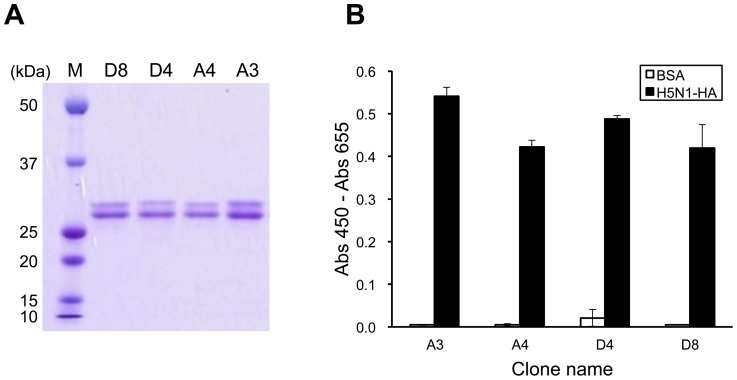
Characterization of soluble Fab fragments. (A) SDS-PAGE of purified Fab fragments. Upper and lower bands correspond to Fd and L chains, respectively. M: Molecular weight standards. (B) ELISA for evaluating binding to H5N1 HA protein. H5N1-HA: recombinant HA from H5N1 A/Vietnam/1194/2004.

Using the pDong system, soluble Fab fragments were expressed in the *E. coli*, and were purified. When the purified Fab fragments were analyzed by SDS-PAGE, two distinct bands with molecular weights of 24 kDa and 26 kDa were observed under reducing conditions, which were identified as the light and Fd immunoglobulin chains (Fig. 3A). Then the antigen-binding activity of Fab fragments was confirmed by ELISA (Fig. 3B). As a result, all the purified Fab fragments showed specific binding to H5N1 HA (A/Vietnam/1194/04), but not to BSA.

**Figure 4:**
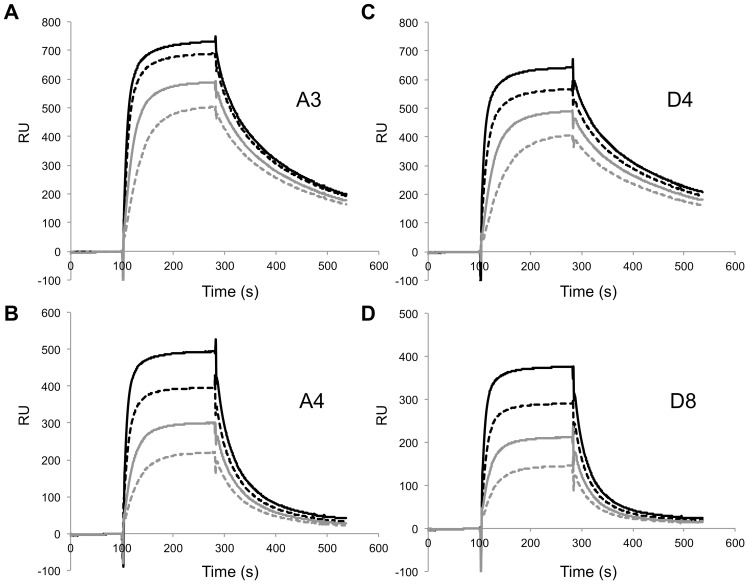
SPR sensorgrams obtained for the purified Fab fragments bound to HA331 peptide. (A) A3, (B) A4, (C) D4, and (D) D8 at 200 (black line), 100 (hatched line), 50 (gray line), and 25 nM (gray hatched line) were applied.

For more quantitative evaluation of antigen binding affinity, SPR analysis was performed. SPR biosensors were utilized to derive the *k*_on_, *k*_off_, and *K*_D_ values for each antibody against the immobilized HA331-derived peptide and H5N1 HA protein ligands. Fig. 4 shows the binding sensorgrams with HA331 peptide, while Fig. S1 shows the assays with H5N1 HA. The association and dissociation rates (*k*_on_ and *k*_off_), as well as the equilibrium dissociation constant (*K*_D =_ *k*_off_/*k*_on_), are summarized in Table 1. For antigen peptides, the *K*_D_ for clones A3 and D4 was much lower than A4 and D8; the *K*_D_ was in the subnanomolar range due to the slower *k*_off_ for these clones. The difference in absolute rate constants observed for the three peptides might at least partly reflect their different immobilization density on the sensorchip surface. On the contrary, the difference in *K*_D_ values for the immobilized HA protein was not obvious between the clones, as it ranged from 36 to 76 nM.

**Table 1:** Kinetic parameters of the Fab fragments obtained by SPR analysis.

Clone	Pep11			Pep11-2				Bio-HA331			H5N1 HA	
	CTGLRNSPQRERRRRKKR			C(PEG_2_)TGLRNSPQRERRRKKR			SAv/Bio-TGLRNSPQRERRRRKKR			A/Vietnam/1194/2004 H5N1 HA		
	*k_a_*	*k_d_*	*K_D_*	*k_a_*	*k_d_*	*K_D_*	*k_a_*	*k_d_*	*K_D_*	*k_a_*	*k_d_*	*K_D_*
	(10^5^/Ms)	(10^−3^/s)	(nM)	(10^5^/Ms)	(10^−3^/s)	(nM)	(10^5^/Ms)	(10^−3^/s)	(nM)	(10^5^/Ms)	(10^−3^/s)	(nM)
A3	13.8	1.11	0.80	7.03	1.62	2.31	6.56	4.85	7.39	0.80	6.09	76.1
A4	7.4	1.92	2.59	4.32	2.18	5.03	4.51	11.00	24.3	1.27	8.77	68.9
D4	21.0	0.98	0.47	8.52	1.38	1.62	5.47	4.13	7.54	1.58	5.66	35.9
D8	11.5	2.92	2.54	6.07	3.49	5.74	3.87	13.50	35.0	1.60	7.86	49.3

XPR36 was used for Pep11 and Pep11-2, and Biacore 2000 was used for the rest.

### The binding of Fab antibodies to highly and low pathogenic H5N1 viruses

To evaluate the potential for the synthesized Fab fragments to bind to viral particles, two H5N1 viruses were prepared. A/Whooper Swan/Mongolia/3/05 (H5N1; H5MNG) is an HPAI H5N1 virus with the multibasic sequence in the HA protein [21]. A/Duck/Hokkaido/Vac-3/07 (H5N1; Vac3) is a low pathogenic H5N1 virus generated by the genetic reassortment of two low pathogenic avian influenza viruses [22]. HA of Vac3 lacks a multibasic sequence, as well as the terminal R of the core epitope (Fig. 2C).

**Figure 5:**
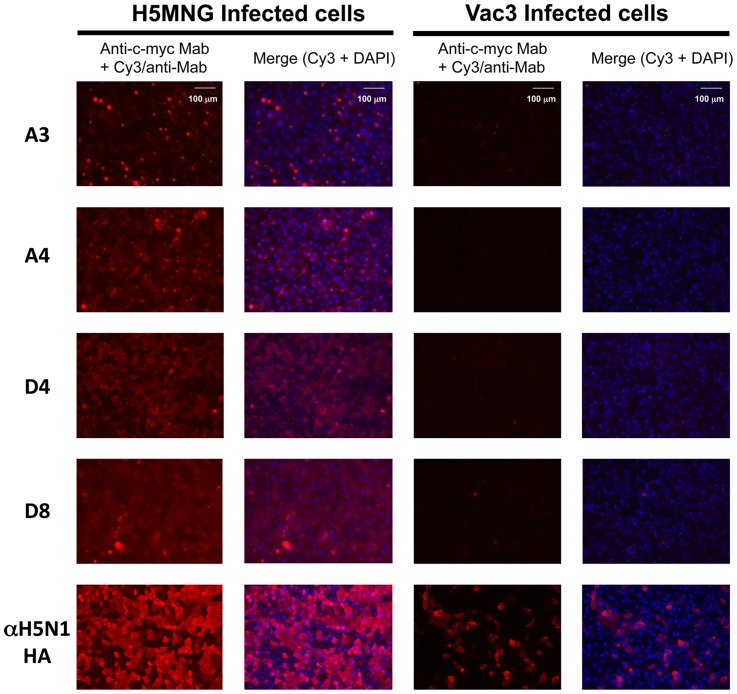
Fab fragment-mediated immunofluorescent staining of Madin-Darby canine kidney (MDCK) cells infected with H5N1 Mongolia virus or Vac (low pathogenic) virus. An anti-H5N1 antibody 9F2E3F3 was also used for staining.

MDCK cells infected with H5MNG or Vac3 were incubated with each Fab fragment and stained with a secondary antibody conjugated to Cy3. Alternatively, the cells were incubated with a control anti-H5N1 HA antibody instead of Fab fragment. When the cells were observed with fluorescent microscopy (Fig. 5), all the Fab fragments stained HPAI H5N1-infected cells. Clearer staining was observed for clones A4 and D4. On the contrary, no staining was observed in the cells infected with the Vac3 virus, except those with a control anti-H5N1 antibody. Thus, the recombinant Fab fragment specifically recognized HPAI H5N1 viral particles containing the HA with multibasic CS.

### Open sandwich ELISA detected HA331 and HPAI H5N1 HA

**Figure 6:**
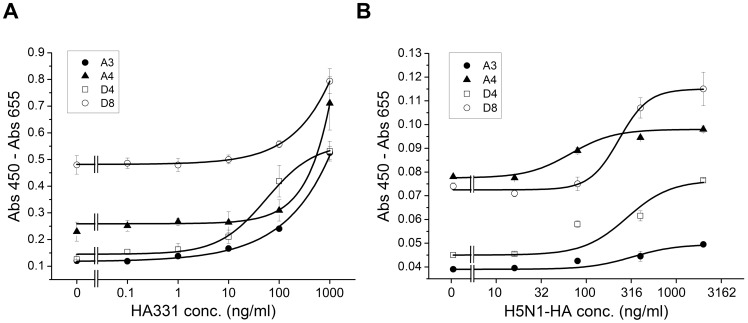
Detection of (A) HA331 peptide and (B) recombinant H5N1 HA protein by open sandwich phage ELISAs. The antigen-dependency of the V_H_/V_L_ interaction was measured with the pDong system.

Open sandwich ELISA (OS ELISA) is a novel immunoassay based on the interchain interaction of an antibody variable region, and it can non-competitively detect an antigen in a sample with less labor and time than traditional sandwich ELISA [23]. OS ELISA has been used for small haptens, but to date, there have been limited attempts to detect peptides and proteins. To test our antibodies for use in OS ELISA, phages displaying V_H_ fragments were prepared by removing the CH1 gene in the vector, as described previously [24]. The dose-response curves of OS ELISA for HA331 and H5N1 HA (A/Vietnam/1194/04) are shown in Fig. 6, A and B, respectively. To our surprise, all the clones detected HA331 and HA with reasonable sensitivity and response, while the signals for the latter was lower possibly due to its larger molecular weight. For the detection of HA331, A3 and D4 reached the lower limit of detection (LOD) in the range of 1 to 10 ng/ml, while A4 and D8 were successful at the higher LOD of 10 to 100 ng/ml; these results were consistent with the *K*_D_ values obtained by SPR. Similarly, for the H5N1 HA protein, the estimated LOD was 100 ng/ml.

### Sequences of anti-CS antibodies

Analysis of the nucleotide and amino acid sequences of the four clones indicated a high percentage identity among these antibodies (Fig. S2). Although the differences between the V_L_ CDRs are not obvious, the V_H_ regions are more variable, and this variability might explain the differences in ligand-binding affinities. The clone D4, which showed the highest affinity and sensitivity, contained the most unique sequences in the V_H_ and V_L_ genes, including the framework regions. The germ line sequences of the variable region genes were analyzed using Ig BLAST (NCBI; http://www.ncbi.nlm.nih.gov/igblast/), and the results are shown in Table S1. The most closely related germline genes for the V segment were IGHV2-70 (clone D8) and IGHV2-5 (clones A3, A4, and D4), while those for the D and J segments were D5, D4 and J4. The nearest relative V germline gene for the light chain was KV7-3 for all the clones. In the J segments, clones A3, D4 and D8 were closely related to Jκ7, while Jκ4 was used for A4.

## Discussion

In this study, we generated four specific antibody Fab fragments with reactivity against the CS of HPAI H5N1 HA by the combination of peptide immunization and phage display selection. The obtained Fab clones specifically bound HPAI H5N1 HA and viral particles in ELISAs and IF assays, respectively. The obtained clones all recognize a core sequence (NS)PQRER observed in the CS of Clade 1 HA. As far as we know, this CS sequence is only found in HPAI H5N1 HAs. Mutation at one of these residues results in reduced recognition, especially at the C-terminal R, which is changed to T in Vac-3 virus. This might be the limitation of our assay since some CS of Clade 2 HA such as that of A/chicken/Indonesia/029/09 is NSPQRES, lacking this essential R. However, all the major HPAI H5N1 HAs we tested showed reasonable reactivity. For other subtypes, H1 and H7 HAs were not recognized probably due to the absence of this epitope sequence. It is worth noting that the CS motif of H6 HA is NVPQIETR (Fig. 2C). However, the alteration of essential R to T similar to Vac-3 (underlined) in the epitope makes the recognition of H6 CS by our antibodies highly unlikely.

The merit of isolating recombinant Fab fragments rather than conventional monoclonal antibodies was demonstrated by the feasibility of OS ELISA, which needs only a single epitope to conduct one-step non-competitive detection. From the SPR analysis, nanomolar *K*_D_ values were obtained for both the antigen peptide and the HPAI HA protein. This would be the reason why we could detect the HPAI HA peptide/protein with a good sensitivity in an OS ELISA. To date, the applicability of OS ELISA in the detection of larger proteins has not been demonstrated except one for lysozyme [23]. In this sense, this is the first successful generation of protein-specific OS ELISA. If we choose appropriate peptide epitope, the method will be generally applicable to many other diagnostic targets. Although the developed ELISA might not be sensitive enough to directly detect viral particles, there are several ways that could enhance its sensitivity. For example, phage-based OS immuno-PCR is more sensitive than OS-ELISA [25]. Combination of this method with SHRT-PCR [26] will allow rapid (less than 20 min) and sensitive detection of influenza viruses. Because our antibodies recognize an epitope that are distinct from other flu subtypes, they could be combined with these novel detection technologies to develop rapid, specific and sensitive diagnostic methods for HPAI H5N1.

All HPAI H5N1 viruses in the A/goose/Guangdong/1/96 (H5N1) lineage have multiple basic residues at the HA CS [27], and almost all HPAI H5N1 viruses are characterized by the CS structure. Thus, diagnosis via the CS provides important information about viral pathogenicity. Although CS-specific detection of H5N1 virus by PCR-based method has been reported [28] and sequence analysis of the CS with reverse transcription-PCR is essential for the molecular pathotyping of H5 subtype viruses [29], this is the first immunological CS detection that is potentially applicable to many popular antibody-based diagnostic platforms. Namely, these Fab fragments can be applied to rapid pathotyping assays such as ELISA and immunochromatography. Although almost all the H5 subtype viruses containing a multibasic sequence are highly pathogenic, there are a few exceptions [30]. For example, the A/goose/Guangdong/2/96 strain was co-isolated with A/goose/Guangdong/1/96 from geese in 1996. While both strains have multibasic sequences, A/goose/Guangdong/2/96 cannot replicate in chickens [31]. Nevertheless, the recombinant Fab fragments we report will be useful novel reagents for the rapid and specific detection of emerging highly pathogenic viruses.

## Materials and Methods

### Materials

The *E. coli* XL10-Gold strain for phagemid cloning and amplification was obtained from Agilent (La Jolla, CA, USA). TG-1 and HB2151 bacteria were obtained from GE Healthcare (Tokyo, Japan) and used for phage production and Fab expression, respectively. Recombinant HA proteins derived from influenza A virus H1N1 (A/California/04/09), H5N1 (A/Vietnam/1194/04, A/Anhui/1/05, and A/Turkey/1/05) and H7N7 (A/Netherlands/219/03), and HA-Fc fusion protein (A/Vietnam/1194/2004(H5N1), in which the cleavage site RERRRKKR was mutated to TETR) were purchased from Sino Biological Inc. (Beijing, China), as well as a mouse monoclonal antibody against H5N1. Synthetic 7-mer peptides used for epitope scanning were from Peptide 2.0 Inc. (Chantilly, VA). Restriction and modification enzymes were obtained from Takara-Bio (Shiga, Japan), Toyobo (Osaka, Japan), Roche Diagnostics (Tokyo, Japan), or New England Biolabs (Ipswich, MA). Oligonucleotides were synthesized by Fasmac Co. (Kanagawa, Japan) or Invitrogen (Tokyo, Japan). Other chemicals, reagents and antibodies, unless otherwise indicated, were obtained from Sigma (St. Louis, MO) or Wako Pure Chemicals (Osaka, Japan).

### Immunization of mice with BSA-HA331 conjugate

The peptide for the H5N1 hemagglutinin CS, HA331 (NH_2_-CTGLRNSPQRERRRRKKR-COOH), and its N-terminal biotinylated derivative bioHA331 (Bio-NH_2_-TGLRNSPQRERRRRKKR-COOH) were synthesized by Genscript (Piscataway, NJ). For the coupling of HA331 peptide to BSA, five hundred microliters of BSA (10 mg/ml in 10 mM phosphate buffer, pH 7.4) was mixed with 70 µl of m-maleimidobenzoic acid N-hydroxysuccinimide ester (MBS) (3 mg in 200 µl of dimethylformamide) at 25°C with stirring, followed by gel filtration through a PD-10 column (GE Healthcare) equilibrated with 50 mM sodium phosphate, pH 6.0. One milliliter of filtered BSA/MBS was added to 100 µl of HA331 solution (4 mg/ml in H_2_O), incubated for 3 h at room temperature, and purified with a PD-10 column equilibrated with phosphate-buffered saline (PBS) (KH_2_PO_4_, 1.47 mM; Na_2_HPO_4_, 8.10 mM; NaCl, 136.89 mM; KCl, 2.68 mM).

Mice were immunized with the BSA-HA331 conjugate by Scrum Co. Ltd. (Tokyo, Japan). In brief, two inbred BALB/c mice were immunized four times at 2-week intervals with 150 µl of 500 µg/ml BSA-HA331 in Freund's complete adjuvant. After the last immunization, blood samples were used to check for the presence of HA331-specific antibodies by an enzyme linked immunosorbent assay (ELISA).

This study was conducted in strict accordance with the recommendations in the Guide for the Care and Use of Laboratory Animals of the National Institutes of Health. The protocol was approved by the Committee on the Ethics of Animal Experiments of the School of Engineering, the University of Tokyo (Permit No. K18-1). All surgery was performed under sodium pentobarbital anesthesia, and all efforts were made to minimize suffering.

### Cloning of antibody variable region genes to construct Fab library

The cDNA fragments for V_H_ and V_L_ were prepared from total RNA derived from the spleen using the PrimeScript One Step RT Kit ver.2 (Takara-Bio) with the manufacturer's protocol and the mouse V_H_/V_L_-specific primers [20]. The 350–400 bp PCR products were purified with a Wizard SV Gel and PCR Clean-Up System (Promega). The V_L_ fragments were then digested with *Sal*I and *Not*I, gel-purified, and ligated into the phagemid pDong1/Fab using T4 DNA ligase at 16°C for 1 h [24]. After confirmation of the inserted V_L_ sequence in several clones, the V_H_ fragments were sub-cloned into the V_L_-inserted phagemid library using restriction enzymes *Sfi*I and *Xho*I. Electro-competent *E. coli* TG-1 cells were transformed with the ligation product and plated on 2YTAG agar (16 g/l tryptone, 10 g/l yeast extract, 5 g/l NaCl, pH 7.2 supplemented with 100 µg/ml ampicillin, 1% glucose, and 1.5% agar) plates overnight at 37°C. The size of the library was estimated from the number of colonies on the plate.

### Fab antibody phage display

*E. coli* TG-1 cells transformed with the phagemid were cultivated in 4 ml of 2YTAG overnight at 37°C. Ten milliliters of 2YTAG was inoculated with 100 µl of the overnight culture and incubated at 37°C with shaking at 200 rpm until the OD_600_ reached ∼0.5, when the helper phage KM13 [32] was added with a multiplicity of infection (MOI) of 20. After incubation at 37°C for 30 min without shaking, the culture was centrifuged at 3,700× g for 15 min. The *E. coli* pellet was resuspended in 50 ml of 2YTAK (2YT medium containing 100 µg/ml ampicillin and 50 µg/ml kanamycin) and incubated overnight with shaking at 30°C. The overnight culture was centrifuged at 10,800× g for 30 min. Ten milliliters of PEG/NaCl (20% polyethylene glycol 6000, 2.5 M NaCl) was added to 40 ml of supernatant, and the mixture was incubated on ice for 1 h. After incubation, the mixture was centrifuged at 6,000× g for 30 min. The pellet was resuspended in 2 ml of PBS and centrifuged at 15,000× g for 10 min to pellet cell debris. The supernatant, containing Fab-displaying phage, was collected.

### Phage ELISA

The microplate (Nunc, Langenselbold, Germany) was coated overnight with 100 µl per well of streptavidin (SAv, 10 µg/ml), BSA (10 µg/ml; Sigma), recombinant influenza A virus HA (0.5 µg/ml), and H5N1 HA fused with Fc (A/Vietnam/1203/2004; 0.5 µg/ml) in PBS at 4°C overnight. After washing, 1 µg/ml bio-HA331 in PBS was added and incubated for 30 min for the SAv-immobilized wells. Each well was blocked at 25°C for 2 h with 2% skimmed milk in PBS (MPBS), washed three times with 0.1% Tween 20 in PBS (PBST), and incubated with 100 µl/well of MPBS containing 10^9^–10^10^ colony forming units (cfu) of Fab-displaying phages at 25°C for 1 h. The plate was washed three times with PBST and incubated with 100 µl/well of HRP-conjugated anti-M13 monoclonal antibody (GE Healthcare) diluted 5000-fold in MPBS at 25°C for 1 h. After three PBST washes, the signal was developed with TMBZ solution [100 µg/ml 3,3′,5,5′-tetramethylbenzidine (Sigma) and 0.04 µl/ml H_2_O_2_ in 100 mM NaOAc, pH 6.0], and the reaction was stopped with 50 µl/well of 10% sulfuric acid. The absorbance was read using a Model 680 microplate reader (Bio-Rad, Tokyo, Japan) at 450 nm with 655 nm as a control.

### Selection of HA331–specific antibody-phage from phage library

Antibody selection from the phage display library was performed on a microplate on which SAv-HA331 was immobilized. After blocking the microplate with MPBS for 2 h, 100 µl of phage solution (10^12^ cfu/ml in PBS) was added and incubated for 1 h at 25°C. After washing 6 times with PBST, phages bound to the microplates were eluted with 100 µl of 1.0 mg/ml TPCK-treated trypsin (Sigma) in PBS. The eluted phage solution was used to infect *E. coli* TG-1 cells for further selection of phage.

Polyclonal and monoclonal phage ELISAs were performed as described above after three rounds of biopanning. The nucleotide sequences of positive clones were analyzed with a Beckman CEQ-8000 DNA sequencer and Genetyx software (Genetyx, Tokyo, Japan).

### Epitope scan

The epitope of Fab was analyzed by phage ELISA for the inhibition by synthetic 7-mer peptides. The SAv-HA331 immobilized well was incubated with 100 µl/well of MPBS containing 10^9^ cfu of Fab-displaying phages added with 20 µg/ml of each peptide at 25°C for 1 h. Sample without peptide was set as a control. The detection was performed as described above.

### Expression and purification of Fab fragments

Soluble Fab fragments of positive antibodies were expressed with a non-suppressing strain HB2151 as described [24]. The His-tagged Fab fragments were purified from the periplasmic and supernatant fractions with Talon Co^2+^-immobilized resin (Clontech, Takara-Bio) according to the manufacturer's instructions. Because single step purification was not sufficient, Fabs were further purified with an Anti-Flag M2 affinity gel (Sigma) according to the manufacturer's instructions. The purified Fabs were analyzed by SDS-PAGE and then used for further analysis.

### Surface Plasmon Resonance (SPR) analysis

The antigen-binding activity of purified Fab fragments was evaluated using SPR biosensors XPR36 (Bio-Rad) and Biacore 2000 (GE Healthcare) at 25°C. A/Vietnam/1194/2004 H5N1 HA (2.5 µg/ml) or SAv (5 µg/ml) in 10 mM sodium acetate, pH 4.5, was injected to immobilize HA (8600 RU) or SAv (1730 RU) on a CM5 sensorchip containing amine coupling reagents. To the SAv-immobilized surface, 1 µM bio-HA331 was injected, and the chips were washed with 1 M NaCl/50 mM NaOH for 1 min each to immobilize HA331 (160 RU). Fab fragments (final concentration 25–200 nM) were applied as analytes at 20 µl/min with HBS-EP (10 mM HEPES, pH 7.4, 150 mM NaCl, 3.4 mM EDTA, 0.05% Tween 20). The sensorgrams for ligand-immobilized and mock-immobilized flow cells were analyzed with BIAevaluation software 4.1 to derive kinetic constants.

### Immunofluorescence

Madin-Darby canine kidney (MDCK) cells (American Type Culture Collection, VA) were maintained in Dulbecco's modified Eagle's medium (DMEM) supplemented with 10% fetal calf serum (FCS) and penicillin-streptomycin. Cells were grown in an incubator at 37°C and 5% CO_2_. The H5N1 influenza virus strains A/Whooper Swan/Mongolia/3/05 (H5MNG) and A/Duck/Hokkaido/Vac-3/07 (Vac3) were grown in 10-day-old embryonated chicken eggs and titrated by plaque assay in MDCK cells.

MDCK cells were inoculated with 4 MOI of H5MNG or Vac3 for 30 minutes and washed with PBS several times. Fresh medium with 10% FCS (without trypsin) was added to each well, and cells were incubated at 37°C. At 6 h post infection, cells were fixed in 4% paraformaldehyde. Fixed cells were treated with each purified Fab for 1 h. Mouse anti-c-myc monoclonal antibody 9E10 (OEM Concepts, Toms River, NJ) was then bound to Fab fragments interacting with HA protein, and these interactions were detected by secondary incubation with Cy3-conjugated anti-mouse antibody (Jackson ImmunoResearch Laboratories, Inc., PA). Cells were observed with fluorescence microscopy (BZ-9000, Keyence, Osaka, Japan).

### Open sandwich ELISA

To display the V_H_ fragment on phages, the pDong1/Fab phagemid was digested with *SgrA*1, whose recognition sites had been incorporated at both ends of the CH1 gene, and the vector was self-ligated to yield pDong/OS. *E. coli* TG1 was transformed with this vector for phage production, and the resultant culture supernatant containing V_H_-displaying phage and the mouse/human chimeric light chain was used for the OS-ELISA. Goat anti-human kappa chain (1 µg/ml in PBS; MBL, Aichi, Japan) was immobilized to plate wells at 4°C for 16 h. After wells were blocked with MPBS, the culture supernatant, together with serially diluted HA331 peptide or H5N1 HA protein, was added to the wells at 25°C for 2 h. After washing, the complex was detected with HRP-conjugated anti-M13 antibody, as described above. Standard curves were made based on the absorbance at each concentration of HA331 or HA protein.

## Supporting Information

Figure S1 **SPR sensorgrams obtained for purified Fab fragments bound to H5N1 HA protein.** The lines are the same as those in Figure 4. H5N1 HA: recombinant A/Vietnam/1194/2004 H5N1 HA.
(PDF)

Figure S2 **Amino acid sequences of the four positive clones.** Complementarity determining regions (CDRs) are shown in bold, and the non-identical positions are boxed.
(PDF)

Table S1 **Comparison of the gene usage for variable regions of the Fab clones.**
(PDF)
