# Supplementary material for: Isolation of Recombinant Phage Antibodies Targeting the Hemagglutinin Cleavage Site of Highly Pathogenic Avian Influenza Virus
Source: PLoS One. 2013 Apr 5;8(4):e61158. doi: 10.1371/journal.pone.0061158 (PMC3618430; doi:10.1371/journal.pone.0061158)
Supplement: Figure S1 — SPR sensorgrams obtained for purified Fab fragments bound to H5N1 HA protein. The lines are the same as those in Figure 4. H5N1 HA: recombinant A/Vietnam/1194/2004 H5N1 HA. (PDF) [file pone.0061158.s001.pdf]

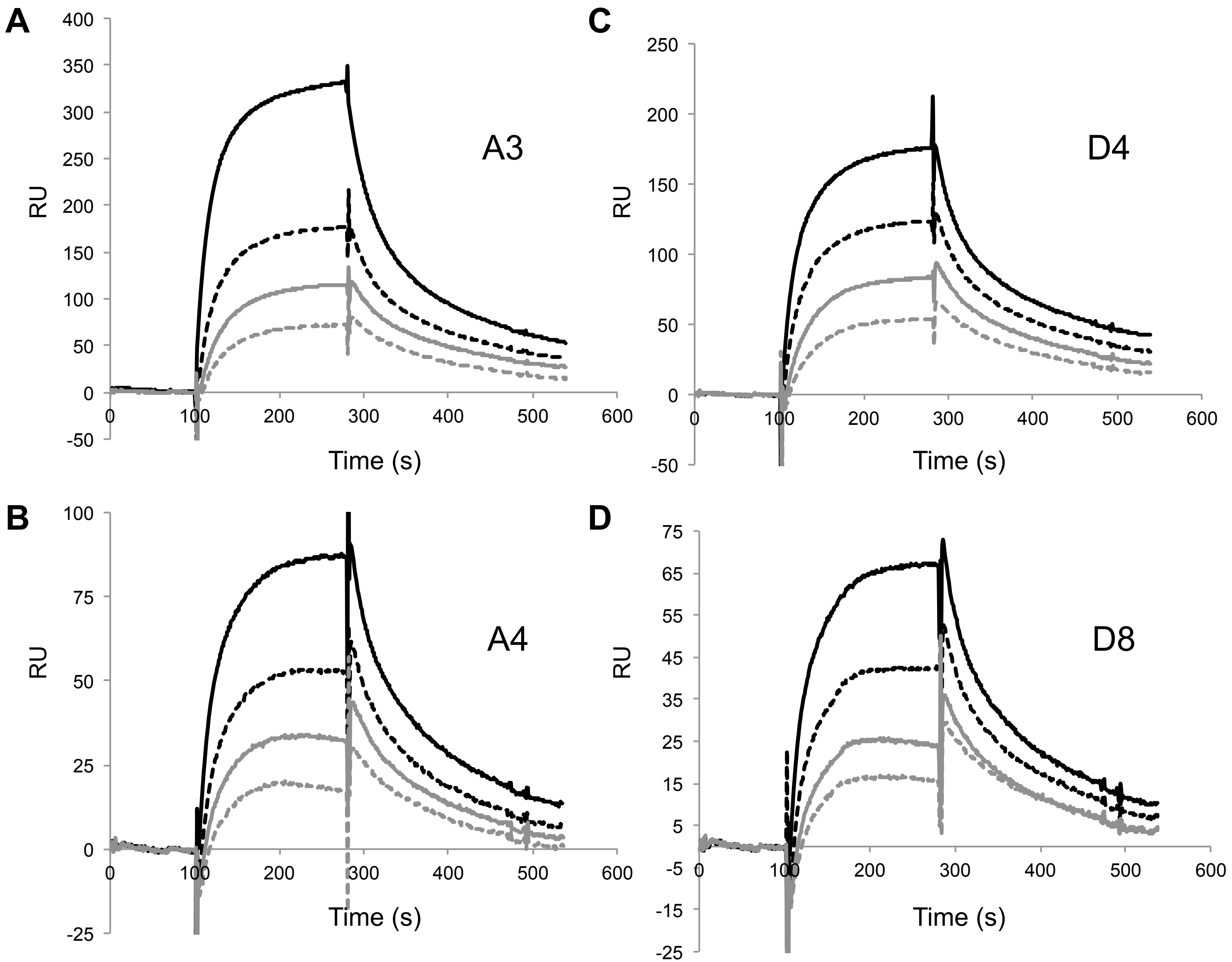

Figure S1. SPR sensorgrams obtained for purified Fab fragments bound to H5N1 HA protein. The lines are the same as those in Figure 4. H5N1 HA: recombinant A/Vietnam/1194/2004 H5N1 HA.
