# Supplementary material for: Isolation of Recombinant Phage Antibodies Targeting the Hemagglutinin Cleavage Site of Highly Pathogenic Avian Influenza Virus
Source: PLoS One. 2013 Apr 5;8(4):e61158. doi: 10.1371/journal.pone.0061158 (PMC3618430; doi:10.1371/journal.pone.0061158)
Supplement: Figure S2 — Amino acid sequences of the four positive clones. Complementarity determining regions (CDRs) are shown in bold, and the non-identical positions are boxed. (PDF) [file pone.0061158.s002.pdf]

A3-VL 1 DIVLTQSPASLAVSPGQRATIFC**KASQSVVYD--GDSYMN**WYQQKPGQPPKLLIY**AASNLES**GIPARFSGSGSGTDFTLNI 80
A4-VL 1 DIVLTQSPASLAMSLGQRATISC**KASQSVVYD--GDSYMN**WYQQKPGQPPKLLIY**AASNLES**GIPARFSGSGSGTDFTLNI 80
D4-VL 1 DIVLTQSPGSLSVSLGQRATISC**KASQSVDYD--GDSYMN**WYQQKPGQPPKLLIY**AASNLES**GIPARFSGSGSGTDFTLNI 80
D8-VL 1 DIVLTQSPASLAMSLGQRATISC**KASQSVVYD--GDSYMN**WYQQKPGQPPKLLIY**AASNLES**GIPARFSGSGSGTDFTLNI 80

A3-VL 81 HPVEEEDAATYYC**QQSSE--DPWT**FGGGTKLEIKRA 117
A4-VL 81 HPVEEEDAATYYC**QQSSE--DPWT**FGGGTKLEIKRA 117
D4-VL 81 HPVEEEDAATYYC**QQSSE--DPWT**FGGGTKLEIKRA 117
D8-VL 81 HPVEEEDAATYYC**QQSSE--DPWT**FGGGTKLEIKRA 117

A3-VH 1 QVTLKESGPGILKPSQTLSLTCSFS**GFSLSTSGMGVG**WIRQSSGKGLEWLA**NIWWDD---DKY**YNPSLKSQLTISKDTSRNQV 83
A4-VH 1 QVTLKESGPGILKPSQTLSLTCSFS**GFSLSTSGMGVG**WIRQPSGKGLEWLA**NIWWDD---DKY**YNPSLKSQLTISKDTSRNQV 83
D4-VH 1 QVTLKESGPGILKPSQTLSLTCSFS**GFSLNTSGMGVG**WIRQPSGKGLEWLA**NIWWDD---DKY**YNPSLKSHLTISKDTSGNQV 83
D8-VH 1 QVTLKESGPGILKPSQTLSLTCSFS**GFSLSTPGMGVG**WIRQPSGKGLEWLA**NIWWDD---DKY**YNPSLKSQLTISKDTSRNQV 83

A3-VH 84 FLKITSVDTADTATYYCAR**RGEYDA------------MDY**WGQGTSVTVSS 132
A4-VH 84 FLKITSVDTADTATYYCAR**RGKYDS------------MDY**WGQGTSVTVSS 132
D4-VH 84 FLKITSVDTADSATYYCAR**RGDYDS------------LDY**WGQGTSVTVSS 132
D8-VH 84 FLKITSVDTTDTATYYCAR**RGNYDA------------MDY**WGQGTSVTVSS 132

Figure S2. Amino acid sequences of the four positive clones. Complementarity determining regions (CDRs) are shown in bold, and the non-identical positions are boxed.
