# Supplementary material for: Isolation of Recombinant Phage Antibodies Targeting the Hemagglutinin Cleavage Site of Highly Pathogenic Avian Influenza Virus
Source: PLoS One. 2013 Apr 5;8(4):e61158. doi: 10.1371/journal.pone.0061158 (PMC3618430; doi:10.1371/journal.pone.0061158)
Supplement: Table S1 — Comparison of the gene usage for variable regions of the Fab clones. (PDF) [file pone.0061158.s003.pdf]

Table S1 Comparison of the gene usage for variable regions of the Fab clones.

Clone name	V segment		D segment		J segment	
	Germ line	Identity (%)	Germ line	Identity (%)	Germ line	Identity (%)
A3-H	V2-5*08	77.3	D5-5*01	100	J4*01	88.6
A4-H	V2-5*08	78.0	D5-12*01	100	J4*01	88.6
D4-H	V2-5*08	76.3	D4-17*01	100	J4*02	88.6
D8-H	V2-70*10	77.7	D4-4*01	100	J4*01	88.6
A3-L	KV7-3*01	78.0	NA*	NA*	J7*02	90.9
A4-L	KV7-3*01	78.0	NA*	NA*	J4*02	88.2
D4-L	KV7-3*01	75.9	NA*	NA*	J7*02	90.9
D8-L	KV7-3*01	77.7	NA*	NA*	J7*02	90.9

*not applicable
